# Sumoylation of Transcription Factor Tec1 Regulates Signaling of Mitogen-Activated Protein Kinase Pathways in Yeast

**DOI:** 10.1371/journal.pone.0007456

**Published:** 2009-10-14

**Authors:** Yuqi Wang, Ameair Abu Irqeba, Mihretu Ayalew, Kristina Suntay

**Affiliations:** Department of Biology, Saint Louis University, St. Louis, Missouri, United States of America; Texas A&M University, United States of America

## Abstract

Tec1 is a transcription factor in the yeast mitogen-activated protein kinase (MAPK) pathway that controls invasive growth. Previously we reported that a fraction of Tec1 protein is sumoylated on residue lysine 54 in normally growing cells. Here we describe regulation and functional consequences of Tec1 sumoylation. We found that activation of Kss1, the MAPK that directly activates Tec1, results in a decrease in Tec1 sumoylation and a concurrent increase of Tec1 transcriptional activity. Consistent with a role of sumoylation in inhibiting Tec1 activity, specifically increasing sumoylation of Tec1 by fusing it to the sumoylating enzyme Ubc9 leads to a dramatic decrease of Tec1 transcriptional activity. Invasive growth is also compromised in Tec1-Ubc9. In contrast, fusing sumoylation-site mutant Tec1, i.e., Tec1^K54R^, to Ubc9 did not significantly alter transcriptional activation and had a less effect on invasive growth. Taken together, these findings provide evidence for regulated sumoylation as a mechanism to modulate the activity of Tec1 and validate Ubc9 fusion-directed sumoylation as a useful approach for studying protein sumoylation.

## Introduction

All cells have the capacity to make appropriate responses to signals perceived from their environment. Mitogen-activated protein kinases (MAP kinases, or MAPKs) coordinate and execute cellular responses to environmental signals [1], [2]. Upon activation by upstream cues, MAP kinases typically enter the nucleus and activate transcription factors to initiate new gene transcription that is required to execute a sequence of events specified by the cues [1]. While the mechanisms by which MAP kinases become activated and inactivated have been well understood, the molecular details by which MAP kinases in turn activate transcription factors are not fully understood [1]. Since ultimately the developmental fate of cells is determined by the activity of transcription factors, a clear understanding of how their activity is regulated by upstream kinase is critical.

The budding yeast has proven to be an appropriate model organism to study the functional interactions between MAP kinases and transcription factors [3], [4]. Indeed, several principles governing the regulation of transcription factors in the MAP kinase pathways were first discovered in this model organism. Prominent examples include the discovery of combinatorial control of transcription factors as a mechanism to achieve signaling specificity [5], [6], the identification of specific transcriptional repressors that keep transcription factors inactive in non-stimulated cells [7], [8] and the more recent demonstration that certain MAP kinases can bind DNA and function as transcriptional regulators [9], [10].

**Figure 1:**
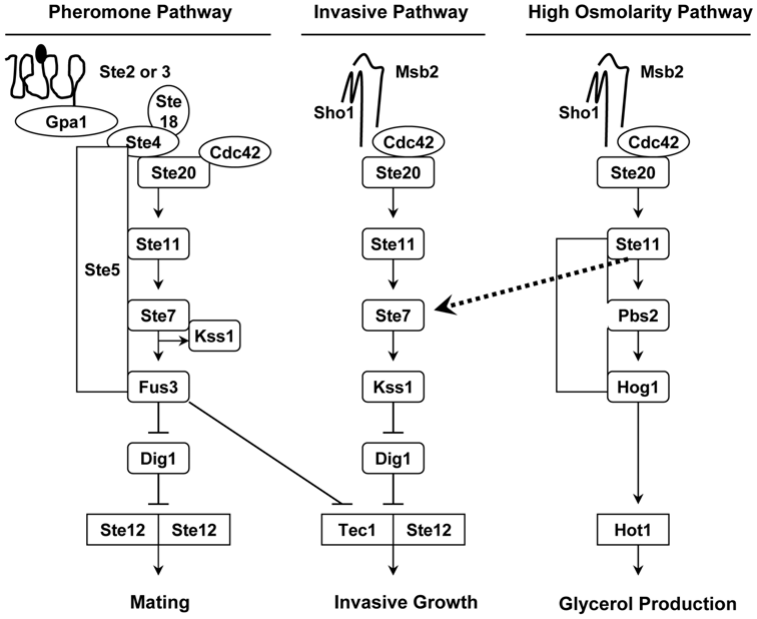
The components of pheromone, invasive and high osmolarity pathways. Adapted from [4], [37]. See text for explanations.

There exist at least four distinct MAP kinase pathways in yeast, each critical for generating a unique biological response [4] (Fig. 1). Specifically, Fus3 is the MAP kinase that mediates the responses of haploid yeast cells to pheromone that is secreted by haploid yeast cells of the opposite mating type [11], [12]. Hog1 is the MAP kinase that becomes activated in response to hyperosmolarity in the environment and promotes the production of internal glycerol to increase internal turgor pressure [13]. Slt2/Mpk1 is the MAP kinase that responds to cell wall stress and plays an important role in maintaining cell wall integrity [14]. Kss1 is the MAP kinase that primarily functions under conditions of nutrient deprivation such as the lack of nitrogen or glucose in the growth media [15]. Under such conditions, activated Kss1 executes a program that leads to the production of cell adhesion molecules, which promote the adherence of yeast cells and thus effectively transform the organism from vegetative to filamentous growth [15]. This pathway is named the invasive or pseudohyphal growth pathway in haploid cells and filamentous pathway in diploid cells. In addition, Kss1 becomes activated in response to pheromone stimulation, but in this case the activation is very transient and is rapidly inactivated by Fus3 via unknown mechanism(s) [16].

Transcription factors that are under the control of Kss1 are Ste12 and Tec1 [4]. Ste12 is unique because it is essential for both the pheromone signaling pathway and the invasive growth pathway [5], [6], [17]. In the pheromone pathway, activation of Fus3 promotes the formation of Ste12-Ste12 homodimer, which binds to promoter regions that contain a DNA sequence named pheromone-response-element (*PRE*) and drives gene expression specifically required for mating [3]. In the invasive growth pathway, activation of Kss1 promotes the formation of a Ste12-Tec1 heterodimer, which binds to filamentation-response-element (*FRE*) and promotes the expression of genes required for invasive growth, such as *FLO11*, whose gene product is an adhesion molecule [15].

Several studies have been carried out to elucidate the mechanisms that regulate the activity of Ste12 and Tec1. It has been shown that two transcriptional repressors, i.e., Dig1/Rst1 and Dig2/Rst2, play important roles in repressing the transcription activity of Ste12 and Tec1 [7], [8]. Some early reports suggest that phosphorylation of these two repressors by activated MAP kinases such as Kss1 somehow leads to de-repression of Ste12 and Tec1, although mutating all six candidate MAP kinase phosphorylation sites on Dig1 did not appear to significantly alter the transcriptional activity of Tec1 [7], [18]. Notably, cells that lack both repressors are still capable of augumenting transcriptional responses from Ste12 [8], indicating the existence of additional mechanism(s) that account for regulation of their activity besides direct repression by Dig1/Rst1 and Dig2/Rst2.

In an earlier effort to elucidate the mechanisms by which Tec1 is regulated, we demonstrated that it is modified by small ubiquitin-like modifer (SUMO) [19]. Here we describe the function and regulation of this sumoylation event. We provide evidence that activation of the upstream kinase Kss1 leads to a suppression of Tec1 sumoylation. Using a newly devised Ubc9-fusion directed sumoylation (UFDS) approach [20], we examined the functional consequences of specific enhancement of Tec1 sumoylation and found that sumoylation has an inhibitory role on Tec1 activity. Together these findings revealed a previously unknown mechanism by which Kss1 controls the transcriptional activity of Tec1 and validated UFDS as a useful approach for studying protein sumoylation.

## Materials and Methods

### Strains and Plasmids

Standard methods for the growth, maintenance, and transformation of yeast and bacteria and for the manipulation of DNA were used throughout. The yeast *S. cerevisiae* strains used in this study are BY4741 (*MAT* ***a*** *leu2Δ met15Δura3Δ*), BY4741-derived mutants lacking *TEC1 and PBS2* (Research Genetics, Huntsville, AL), BY4741-derived mutants lacking both *TEC1* and *PBS2* (*tecl::URA3 pbs2::kanMX*, this work), W303 strain Z1315 (*MAT* ***a*** *ade2-1 trp1-1 can1-100 leu2-3,112 his3-11,15 ura3 STE12::18MYC::TRP1*, from Richard Young, Whitehead Institute at MIT) [21], Z1315-derived mutants lacking *TEC1* (*tec1::URA3*) [21], Σ1278-based invasive strain (*MAT* **a** *leu2Δ ura3Δ*, from Joseph Heitman, Duke University) and Σ1278-derived mutants lacking *TEC1* (*tec1::URA3*).

Expression plasmids used in this study that have been described previously are pRS315-TEC1-3xFLAG, pRS315-TEC1^K54R^-3xFLAG [19]. Plasmids expressing Tec1-Ubc9-3xFlag and Tec1^K54R^-Ubc9-3xFlag were constructed by the following steps. The *UBC9* open reading frame flanked with a *SpeI* site and a *HindIII* site was amplified by PCR and was subcloned to a parent vector pRS315 containing engineered DNA fragment encoding three tandem FLAG epitopes (pRS315-UBC9-3xFLAG). PCR primers were 5′-GAC TAG TAG TAG TTT GTG TCT ACA GCG TC-3′ and 5′-CCC AAG CTT TTT AGA GTA CTG TTT AGC-3′. DNA fragments containing the TEC1 promoter as well as the TEC1 open reading frame were amplified using pRS315-TEC1-3xFLAG and pRS315-TEC1K54R-3xFLAG as templates. SpeI sites were used for subcloning the fragments into pRS315-UBC9-3xFLAG. PCR primers were 5′-GAC TAG TCC ATT TAG TGA CAC AGG TGA GG-3′ AND 5′-GAC TAG TAT AAA AGT TCC CAT GCG ATT GG-3′.

A triple-FLAG epitope tag was placed at the N terminus of Ulp1 (FLAG-ULP1) by PCR amplification and subcloning into the TOPO site of the yeast expression vector pYES2.1/V5-His-TOPO (Invitrogen) (2 µm, *URA3, GAL1* promoter). PCR primers were 5′-CGG AAT TCC AGA ATG GAT TAT AAA GAT GAC GAT GAC AAG GGT ATG TCA GTT GAA GTA GAT AAG-3′ and 5′-CTA TTT TAA AGC GTC GGT TAA -3′.

### Growth, Transcription, Phosphorylation, and Degradation Bioassays

Growth and reporter-transcription assays were conducted as described previously [19]. Phosphorylation of Fus3, Kss1 and Mpk1/Slt2 were monitored by immunoblotting of whole cell extracts, following the same procedures described previously [22]. Briefly, mid-log cell cultures were grown on appropriate medium, and treated or not treated with 0.5 M KCl for the indicated length of time. Growth was stopped by the addition of 10 mM NaN_3_ and transfer to an ice bath. Cells were washed and resuspended directly in boiling SDS-PAGE sample buffer (62.5 mM Tris-HCl, pH 6.8, 10% glycerol, 2% SDS, 1% 2-mercaptoethanol, 0.0005% bromphenol blue) for 10 min, subjected to glass bead homogenization, and clarified by microcentrifugation. Following SDS-polyacrylamide gel electrophoresis and transfer to nitrocellulose, the membrane was probed with antibodies to phosphor-p44/42 at 1∶1,000, phosphor-p38 at 1∶1,000 (from Cell Signaling), Hog1 at 1∶200 (from Santa Cruz), and Pgk1 at 1∶75,000 (from Jeremy Thorner, University of California, Berkeley, CA). Immunoreactive species were visualized by enhanced chemiluminescence detection (Pierce) of horseradish peroxidase-conjugated anti-rabbit IgG (Bio-Rad) or anti-goat IgG (Santa Cruz Biotechnology). Specificity of detection was established using *mpk1Δ*, *fus3Δ*, *kss1Δ* and *hog1Δ* cell extracts as negative controls.

### Immunoprecipitation

Sumoylation of Tec1 was examined by immunoprecipitation of Flag-tagged Tec1 and its variants, i.e., Tec1^K54R^, Tec1-Ubc9 fusion, and Tec1^K54R^-fusion. Immunoprecipitates were probed with antibodies against SUMO (from Stefan Jentsch, Max Planck Institute of Biochemistry, Germany), Flag (from Sigma) and Ubc9 (from Santa Cruz). Interaction between Tec1 and Ste12 were examined by immunoprecipitation of Flag-tagged Tec1 and its variants and immunoblotting with Myc antibodies (from Henrik Dohlman, University of North Carolina, Chapel Hill, NC) for the detection of Myc-tagged Ste12. Cells (100 ml) transformed with appropriate expression plasmids were grown to *A*_600__nm_ ∼1, treated with 0.5 M KCl if indicated, harvested, and resuspended in 550 µl of lysis buffer (50 mM NaPO_4_, pH 7.5, 400 mM NaCl, 0.1% Triton X-100, 10% glycerol, 0.5 mM dithiothreitol, 25 mM NaF, 25 mM glycerophosphate, 1 mM sodium orthovanadate, 10 mM *N*-ethylmaleimide, 5 mM phenylmethylsulfonyl fluoride, and one pellet of complete EDTA-free protease inhibitor mixture (Roche Applied Science)). This and all subsequent manipulations were carried out at 4°C. Cells were subjected to glass bead vortex homogenization for 30 s, repeated 8 times, and centrifuged twice at 6000×*g* for 5 min and 25 min. Lysates were incubated for 2 h with a bead volume of 10 µl of anti-FLAG M2 affinity resin (Sigma) equilibrated in lysis buffer. Immunoprecipitates were collected by centrifugation at 1000×*g* for 30 s, and pellets were washed with 1 ml of lysis buffer for 3 min, repeated 4 times, before final resuspension in 30 µl of 2x SDS-PAGE sample buffer. Each sample was resolved by 7.5% polyacrylamide gel electrophoresis and immunoblotting with appropriate antibodies as indicated.

## Results

MAP kinases play important roles in many biological processes. Although MAP kinases can phosphorylate a range of cytosolic substrates to execute their functions, their main effects in regulating cell development are achieved via activation of specific transcription factors. Thus understanding how transcription factors are controlled by MAP kinases is critical for a clear elucidation of MAP kinase signaling mechanisms. Previously, we demonstrated that Tec1, a transcription factor controlled by a MAP kinase Kss1 in yeast, is modified by a small ubiquitin-like modifier (SUMO) [19]. We were interested in whether the modification is regulated by the activation status of Kss1 and what functional roles sumoylation of Tec1 may have in regulating signaling outcome of the Kss1-mediated invasive pathway. It has been technically difficult to address these questions however, partly due to a lack of a stimulus that can be conveniently used to specifically stimulate Kss1 but not any other MAP kinases. Consequently, whether sumoylation could serve as a mechanism for Kss1 to modulate the transcriptional activity of Tec1 is still unknown.

**Figure 2:**
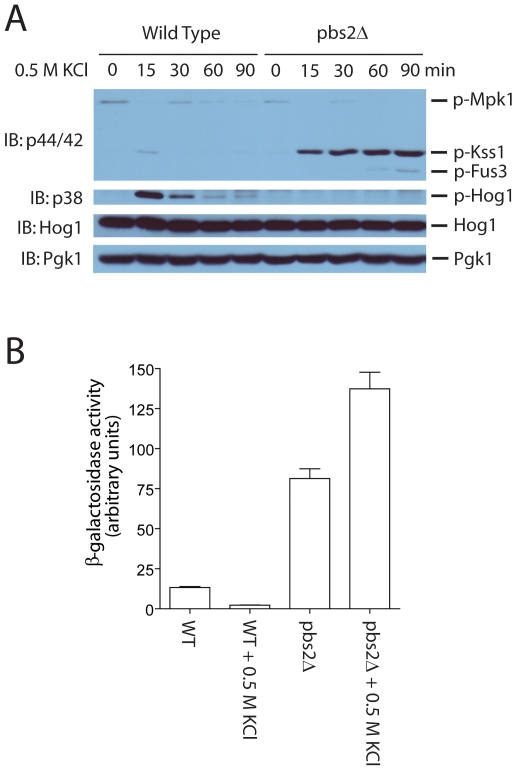
Hyperosmolarity induces specific activation of Kss1 in the *pbs2*Δ mutants. *A*, hyperosmolarity-induced activation of MAP kinases of wild type and *pbs2Δ* mutants was measured by comparing phosphorylation of Mpk1/Slt2, Kss1, Fus3 and Hog1. Whole cell extracts were prepared from wild type and *pbs2Δ* mutants treated with 0.5 M KCl for the indicated time, resolved by 10% SDS-PAGE, and probed with anti-phospho-p42/44 (*top*) or anti-phospho-p38 (*top middle*) or anti-Hog1 (*bottom middle*) as well as anti-Pgk1 (*bottom*) antibodies to confirm equal loading of each *lane*. IB, immunoblotting. *B*, transcription activity was measured in wild type and *pbs2Δ* mutants transformed with a plasmid containing an invasive-specific *FRE* reporter (*TEC1* promoter, lacZ reporter) and treated or not treated with 0.5 M KCl for one hour. *Error bars*, ± S.E. The data shown are representative of three independent experiments performed in triplicate.

To obtain a system in which Kss1 is the only MAP kinase that is predominantly activated, we utilized the substantial component sharing between the Kss1-mediated invasive pathway and the Hog1-mediated high-osmolarity pathway [23] (Fig. 1). In response to an increase in osmolarity, cells activate an enzyme cascade that leads to activation of Ste11, a MAP kinase kinase kinase which is the direct upstream kinase of both Ste7 in the Kss1-mediated invasive pathway and Pbs2 in the Hog1-mediated high-osmolarity pathway. It has been shown that removal of Pbs2 can lead to specific activation of Ste7 and consequently activation of Kss1 by hyperosmolarity stimulation (Fig. 1) [18], [24], [25]. We were interested in whether treating the *pbs2Δ* cells can achieve *specific* activation of Kss1. To test this, we examined the activation status of all four MAP kinases (i.e., Fus3, Kss1, Hog1, and Mpk1) in wild type versus the *pbs2Δ* cells upon treatment of 0.5 M KCl, using phosphor-specific antibodies directed against dually phosphorylated p44/42 and p38 MAP kinases. As shown previously, in wild type cells, salt treatment induces very weak and transient activation of Kss1 [25], [26]. However, the same treatment leads to a dramatic and prolonged Kss1 activation in the *pbs2Δ* cells (Fig. 2A). Importantly, Kss1 is the only MAP kinase that is markedly activated under this condition. Hog1 is not activated due to the lack of its upstream kinase Pbs2; basal phosphorylation of Mpk1/Slt2 is rapidly decreased as reported previously [27], and Fus3 is only weakly activated at later time points.

To examine whether salt-induced activation of Kss1 in the *pbs2Δ* cells leads to an increase of Ste12-Tec1 transcription activity, we then measured transcription induction using a *FRE* promoter fused to *lacZ* [5]. Compared to wild type, the *pbs2Δ* cells exhibited an elevated basal *FRE*-*lac*Z activity, and importantly the activity was further augumented by salt treatment (Fig. 2B), indicating that salt-induced activation of Kss1 in the *pbs2Δ* cells is capable of enhancing the activity of its downstream transcription factors.

**Figure 3:**
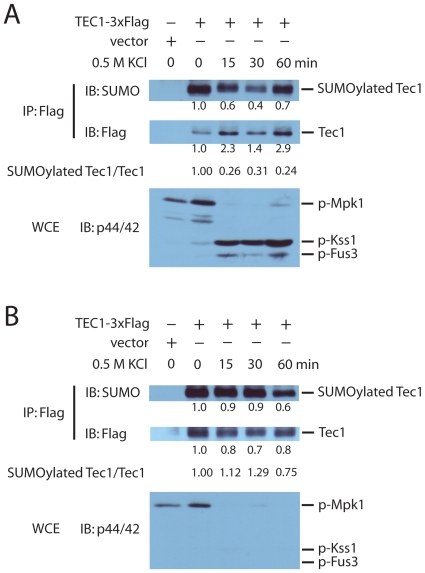
Specific activation of Kss1 but not increased osmolarity *per se* suppresses sumoylation of Tec1. *A*, *tec1Δpbs2Δ* cells transformed with triple-FLAG-tagged Tec1 or the parent vector were grown and treated or not treated with 0.5 M KCl for the indicated time. Whole cell lysates were prepared and were immunoprecipitated with M2 anti-FLAG resin and subjected to immunoblotting using anti-SUMO or anti-FLAG antibodies (upper panels). Numbers under each band refer to the difference in band intensity relative to lane 2, as determined by scanning densitometry. The quantification of SUMOylated Tec1/Tec1 was calculated by dividing the relative band intensity of sumoylated Tec1 by that of non-sumoylated Tec1. IP, immunoprecipitation; IB, immunoblotting; WCE, whole cell extract. *B*, the experiments were conducted exactly the same as shown in panel A, except that *tec1Δ* but not *tec1Δpbs2Δ* cells were used for transformation. The data shown are representative of at least two independent experiments.

Having confirmed salt-treatment of the *pbs2Δ* cells as an appropriate approach to achieve specific activation of Kss1 (and to a less extent, Fus3), we sought to determine whether the activation status of Kss1 regulates sumoylation of Tec1. For this purpose, we immunopurified Tec1 tagged with a triple FLAG epitope and probed the purified samples by immunoblotting with antibodies to FLAG as well as to SUMO. This approach has been successfully used in our previous study to demonstrate sumoylation of Tec1 [19]. Interestingly, treatment by 0.5 M KCl led to a rapid and substantial inhibition of Tec1 sumoylation, as evidenced by a more than three-fold decrease in the ratio of sumoylated Tec1 versus non-sumoylated Tec1, about 15 minutes after salt treatment (Fig. 3A). The decrease of sumoylation correlated very well with an increase of Kss1 activity, as revealed by immunoblotting with antibodies against phosphor-Kss1 (Fig. 3A), suggesting an inhibitory role of Kss1 on sumoylation of Tec1. It is possible that the rapid inhibitory effect of salt treatment on Tec1 sumoylation is purely due to an alteration of osmolarity *per se* and would occur with or without Kss1 activation. In that case, treatment of wild type cells instead of the *pbs2Δ* mutant with the same concentration of salt should similarly repress Tec1 sumoylation. To test this, we examined Tec1 sumoylation in wild type cells upon salt treatment. As shown in Fig. 3B, during the first half hour of salt treatment, where we saw significant repression of Tec1 sumoylation in the *pbs2Δ* mutants, there was no decrease in the relative level of sumoylation in the wild type cells. Since Kss1 is not significantly activated in wild type cells (Fig. 2A & Fig. 3B), the effect we observed in the *pbs2Δ* cells is due to activation of Kss1 and not because of an alteration of osmolarity *per se*.

Given the stimulatory effect of Kss1 on the transcriptional activity of Tec1 and its inhibitory role on the sumoylation status of Tec1, we reasoned that sumoylation of Tec1 in normal growing cells might serve to repress its transcriptional activity. To test this model, we sought to examine the effects of enhancing Tec1 sumoylation. A prediction is that enhancement of Tec1 sumoylation would lead to further repression of Tec1 transcriptional activity and consequently would impair invasive growth. In order to specifically enhance sumoylation of Tec1 but not any other SUMO targets, we employed a recently developed approach named Ubc9 fusion directed sumoylation (UFDS) [20], [28]. In this approach, a fusion protein between a substrate and the SUMO conjugating enzyme Ubc9 is used to direct sumoylation of the substrate. This approach has been applied to a few well-characterized SUMO substrates in mammalian cells including p53 [20]. It has been demonstrated that enhanced sumoylation brought about by UFDS occurred mainly on the authentic sumoylation sites of the substrates [28].

**Figure 4:**
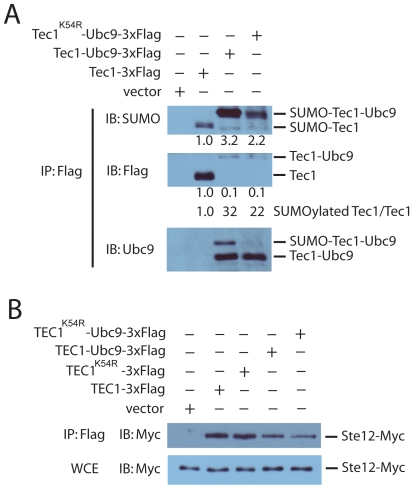
Ubc9 fusion-directed sumoylation (UFDS) of Tec1. *A*, whole cell lysates from *tec1Δ* cells transformed with plasmids that express triple-FLAG-tagged Tec1, a Tec1-Ubc9 fusion, a Tec1^K54R^-Ubc9 fusion, or the parent vector were immunoprecipitated with M2 anti-FLAG resin and subjected to immunoblotting using anti-SUMO, anti-FLAG, or anti-Ubc9 antibodies. IP, immunoprecipitation; IB, immunoblotting; SUMO, small ubiquitin-like modifier. Numbers under each band refer to the difference in band intensity relative to lane 2, as determined by scanning densitometry. The numbers of SUMOylated Tec1/Tec1 were calculated by dividing relative band intensity of sumoylated Tec1 by that of non-sumoylated Tec1. *B*, *tec1Δ* cells containing 18-Myc-STE12 at its genomic locus were transformed with either an empty vector or a plasmid that expresses triple-FLAG-tagged Tec1, Tec1^K54R^, a Tec1-Ubc9 fusion, or a Tec1^K54R^-Ubc9 fusion. Whole cell extracts were immunoprecipitated with M2 anti-FLAG resin and the levels of co-immunoprecipitated Ste12 were detected by immunoblotting using anti-Myc antibodies. The levels of 18-Myc-Ste12 in the applied whole cell extracts were similarly detected by immunoblotting using anti-Myc antibodies. The data shown are representative of at least two independent experiments.

To examine whether UFDS is a valid approach for studying Tec1 sumoylation, we made fusion protein consisting of Tec1 and Ubc9. A similar fusion between the sumoylation site mutant Tec1^K54R^ and Ubc9 was constructed as a control to assess whether UFDS directed sumoylation occurs on the known endogenous sumoylation site on Tec1 [19]. We then examined sumoylation of fusion proteins via immunopurification and immunoblotting with antibodies against SUMO, FLAG, as well as Ubc9. As revealed by the SUMO blot, sumoylation of Tec1-Ubc9 fusion is dramatically increased as compared to wild type (Fig. 4A). The ratio of signals from sumoylated proteins versus non-sumoylated proteins (the Flag blot) increased even more in the Tec1-Ubc9 fusion. Importantly, sumoylation of Tec1-Ubc9 mainly occurs on the authentic sumoylation site Lys54 of Tec1, as the sumoylation signal is much diminished in Tec1^K54R^-Ubc9 mutant (Fig. 4A). This is especially apparent in the blot probed with Ubc9 antibody that can detect both sumoylated and non-sumoylated species of the fusion proteins (Fig. 4A).

Having determined that fusion of Tec1 and Ubc9 enhances sumoylation of Tec1 at Lys54, we sought to examine the functional consequences of increasing sumoylation of Tec1. One common function of sumoylation is regulating protein-protein interactions [29]. Tec1 requires dimerization with Ste12 to be functional, thus it is possible increasing sumoylation of Tec1 might impact its interaction with Ste12. To test this, we immunopurified Flag-tagged Tec1-Ubc9, and Tec1^K54R^-Ubc9 fusion proteins and probed the purified samples for co-purified Ste12. For immuno detection of Ste12, the cells used for immunoprecipitation also expressed an N-terminal poly-Myc tagged version of Ste12 (18-Myc-Ste12). 18-Myc-Ste12 was used because the multiple epitopes enhance detection and it has been shown previously that the tag does not affect activity of Ste12 [21]. Both fusion proteins were able to pull down Ste12, and there was no decrease in the amount of Ste12 that co-purified with Tec1-Ubc9 as compared with Tec1^K54R^-Ubc9 (Fig. 4B). These data indicate that increased sumoylation displayed by Tec1-Ubc9 does not alter its interaction with Ste12.

**Figure 5:**
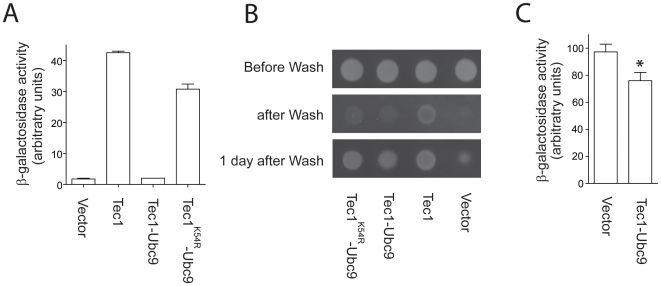
Specific enhancement of Tec1 sumoylation represses Tec1 activity. *A*, transcription activity was measured in *tec1Δ* cells co-transformed with plasmids that express triple-FLAG-tagged Tec1, a Tec1-Ubc9 fusion, a Tec1^K54R^-Ubc9 fusion, or the parent vector and a plasmid containing an invasive-specific *FRE* reporter (*TEC1* promoter, lacZ reporter).*B*, the same cells as in *panel A* were spotted onto solid YPD medium and after 2 days rubbed vigorously under a stream of water to detect invasive growth. *C*, transcription activity was measured in wild type cells transformed with plasmids that express a Tec1-Ubc9 fusion or the parent vector and a plasmid containing an invasive-specific *FRE* reporter as described in *panel A*. The data were statistically analyzed by *t* test with a *p* value of <0.05. *Error bars*, ± S.E. The data shown are representative of at least two independent experiments performed in triplicate.

We then examined whether signaling output is affected by increased sumoylation of Tec1. For this purpose, we first measured the transcriptional activity of Tec1-Ubc9 using the *FRE-lacZ* reporter as before. Strikingly, Tec1-Ubc9 exhibits nearly no *FRE-lacZ* activity (Fig. 5A). The lack of transcriptional activity of Tec1-Ubc9 apparently is not due to the fusion of Ubc9, as a similarly constructed fusion protein Tec1^K54R^-Ubc9 has only slightly decreased *FRE-lacZ* activity as compared to wild type Tec1. To examine whether inhibition of *FRE* transcription by Tec1-Ubc9 has any biological consequence, we compared the invasive growth of Tec1, Tec1-Ubc9 and Tec1^K54R^-Ubc9. Consistent with the results from transcription assays, invasive growth is significantly diminished in Tec1-Ubc9 cells but was decreased to a less extent in Tec1^K54R^-Ubc9 cells (Fig. 5B). To examine whether Tec1-Ubc9 has any dominant-negative effect on wild type Tec1, we compared the *FRE-lacZ* activity in wild type cells that express either an empty vector or Tec1-Ubc9. Interestingly, Tec1-Ubc9 displayed a slightly inhibitory effect on wild type Tec1 (Fig. 5C). Taken together, these findings provide evidence that enhanced sumoylation of Tec1 diminishes its transcriptional activity and consequently impairs invasive growth.

## Discussion

In this report, we examined the regulation and role of sumoylation of Tec1, a transcription factor in the MAP kinase signaling pathway that controls invasive growth in haploid yeast cells. By taking advantage of the unique behavior of *pbs2Δ* cells, which display hyperosmolarity-stimulated specific activation of Kss1, we demonstrated that Tec1 sumoylation is regulated by Kss1. Specifically, we demonstrated that activation of Kss1 leads to a significant decrease in Tec1 sumoylation. Using the recently developed UFDS (Ubc9 fusion dependent sumoylation) strategy, we also showed that specifically enhancing Tec1 sumoylation dramatically inhibits its activity. Together, our study provides evidence that the Kss1-regulated decrease of Tec1 sumoylation serves as a mechanism for activating Tec1.

What could be the mechanism by which Kss1 regulates the sumoylation level of Tec1? One possibility is that Kss1 can directly phosphorylate Tec1 and phosphorylation targets Tec1 for desumoylation. It has been demonstrated previously that Tec1 can be phosphorylated by Fus3, the MAP kinase of the pheromone signaling pathway [30], [31], [32], but whether Tec1 is also a substrate of Kss1 remains to be determined. Another possibility is that Kss1 might have a regulatory role for the machinery that controls Tec1 sumoylation *in vivo*. It is not without precedent that a MAP kinase can regulate the activity of enzymes critical for ubiquitin and ubiquitin-like modifications. For instance, it has been reported previously that JNK can regulate the activity of an E3 ubiquitin ligase Itch [33]. The possibility that Kss1 may inhibit the main components of the sumoylation pathway such as Ubc9 is unlikely, however. Under the same condition that we detected stimulus-dependent decrease of Tec1 sumoylation, the global level of protein sumoylation is increased (Irqeba and Wang, unpublished observation), indicating that Kss1 does not have a general role of inhibiting protein sumoylation.

One often utilized strategy for studying the function of protein sumoylation is determining the consequences of diminishing or enhancing the sumoylation level of the protein. Identifying and mutating the acceptor lysine residues is one of the commonly used approaches for blocking sumoylation. However, certain limitations are associated with this approach. For instance, a number of modifications such as ubiquitination, acetylation and methylation can occur on lysine residue [34]. Therefore it is not necessarily appropriate to attribute the phenotype of a sumoylation site mutant solely to a change in sumoylation. A complementary approach is to examine the consequence of enhancing the sumoylation level of the protein. Inactivating desumoylating enzyme(s) is commonly used for that purpose. However, sumoylation of many substrates would be affected by this approach, since there exist only very limited numbers of desumoylating enzymes. For instance, in budding yeast, Ulp1 and Ulp2 are the only two known desumoylating enzymes [35], [36], and Ulp1 is responsible for most of the desumoylating events. Inhibiting Ulp1 will increase the sumoylation level of many substrates in addition to Tec1. The recently developed Ubc9-fusion dependent sumoylation (UFDS) overcomes the limitation, and can be used to specifically enrich the sumoylation of a specific substrate [28]. Using this approach, we showed that specifically enhancing Tec1 sumoylation dramatically inhibits its transcriptional activity. The behavior of Tec1-Ubc9 and Tec1^K54R^-Ubc9 provided some useful insights as to how sumoylation may inhibit Tec1 activity. Since nearly the same amounts of non-sumoylated species of Tec1-Ubc9 and Tec1^K54R^-Ubc9 are present in the cells, the dramatically different signaling phenotypes of cells that express Tec1-Ubc9 and Tec1^K54R^-Ubc9 must originate from the sumoylation of Tec1-Ubc9. This notion is supported by our data that Tec1-Ubc9 can dominantly inhibit Tec1 activity.

How would sumoylated Tec1-Ubc9 inhibit transcription? One possibility is that it recruits transcriptional repressors to the promoter regions that are controlled by Tec1. To test this possibility, we examined the signaling behavior of Tec1-Ubc9 in cells that lack known Tec1 repressors Dig1 and Dig2. However, the inhibitory effect of Tec1-Ubc9 on signaling cannot be relieved by deletion of either *DIG1* or *DIG2* genes (data not shown). It is still possible that other more general transcriptional repressors such as histone deacetylases might be recruited. Future work will be directed to identify the proteins that might specifically interact with sumoylated Tec1-Ubc9, and to test whether these unknown proteins might play important roles in determining the signaling output of invasive pathways.

Ste12 is also sumoylated and its sumoylation is stimulated by pheromone treatment, a condition that activates both Fus3 and Kss1 [19]. Thus the regulation of Ste12 sumoylation appears to be different from that of Tec1. It would be interesting to understand why sumoylation of these two related transcriptional factors are regulated differently by their upstream kinases. Distinct from Tec1, the principle sumoylation site on Ste12 has not been identified yet [19]. Future work should be directed to identify Ste12 sumoylation site(s) and examine the functional consequences of inhibiting Ste12 sumoylation (via Lys-to-Arg mutation of the sumoylation site). Conceivably, once the sumoylation site on Ste12 is identified, the UFDS approach could also be applied to examine the functional consequences of Ste12 sumoylation.
